# Supplementary material for: In Silico Evaluation of Sesquiterpenes and Benzoxazinoids Phytotoxins against Mpro, RNA Replicase and Spike Protein of SARS-CoV-2 by Molecular Dynamics. Inspired by Nature
Source: Toxins (Basel). 2022 Aug 29;14(9):599. doi: 10.3390/toxins14090599 (PMC9506577; doi:10.3390/toxins14090599)
Supplement: Supplementary file 1 [file toxins-14-00599-s001.zip › toxins-1878158-supplementary.pdf]

Table S1. RMSD values of tested compounds against M^{pro} of SARS-CoV-2.

M^{pro} SARS-CoV-2								
**RMSD in Cluster**	**1**	**2**	**3**	**4**	**5**	**6**	**7**	**8**
Azithromycin	0	0.13	0.44					
Hydroxychloroquine	0	0.64	1.81	1.1				
Favipiravir	0	0.07	0.23	0.23	0.4			
Artemisinin	0	0.11	0.53	0.31	1.11	1.1		
Met-4F-Benzo	0	0.3	0.45	1.74	1.42			
Cynaropicrin	0	1.45	1.86	1.98				
Fluor-Cynaro	0	0.14	0.35					
Costunolide	0	0.02	0.06					
DHC	0	0.06	0.61	0.67				
Reynosin	0	0.18	0.17	0.21	0.18			
Santamarine	0	0.03	0.06	0.27	0.58			
Fluor-Reynosin	0	1.5	1.83					
Fluor- Santamarine	0	1.82	0.42	0.09				
Alanto	0	1.47	1.56	1.32				
Alpha-Cyclo	0	0.12	0.13	0.49				
Beta-Cyclo	0	0.06	0.64	0.78				
3-DeBra	0	0.04	0.08	0.07				
APO	0	0.53	0.48	0.38	0.41	0.86	0.41	
DisOH	0	1.55	1.7	1.64				
DisNH2	0	1.63	1.96	1.79	1.86	1.9		
Fluor-APO	0	0.04	1.78					
Fluor-DisOH	0	0.3	1.69					
Fluor-DisNH	0	0.8	0.73					
DIBOAa	0	0.09	0.09					
DIBOAb	0	0.02	0.05	0.19				
DIMBOAa	0	0.02	0.33					
DIMBOAb	0	1.53	1.49					
DDIBOA	0	0.05	0.32	0.1	0.44			
6Cl-DDIBOA	0	0.09	0.08	0.69	1.65	1.58		
6F-DDIBOA	0	0.14	0.1	4	1.71			
8Cl-DDIBOA	0	0.39	0.17	0.65	0.69	0.81	0.75	1.97

Table S2. RMSD values of tested compounds against RNA Replicase of SARS-CoV-2.

**RNA Replicase SARS-CoV-2**									
**RMSD in Cluster**	**1**	**2**	**3**	**4**	**5**	**6**	**7**	**8**	**9**
Azithromycin	0	1.86	0.21						
Hydroxychloroquine	0	1.02	1.85						
Favipiravir	0	0.37	0.24	0.42	0.66	0.67	1.67		
Artemisinin	0	0.02	1.27	1.26	1.26	1.28	1.27	1.21	1.16
Met-4F-Benzo	0	0.15	0.77	0.99	0.92	0.82			
Cynaropicrin	0	1.75	1.87	0.33	1.42	1.66	1.76	1.89	
Fluor-Cynaro	0	1.63	1.68						
Costunolide	0	0.5	0.45	1.5					
DHC	0	0.37	0.03	0.3	1.94				
Reynosin	0	0.28	0.31	0.19	0.25	0.13	1.16	1.66	
Santamarine	0	1.37	1.9	1.92	1.78	1.88			
Fluor-Reynosin	0	0.19	1.74	1.6	1.3	1.6	0.84	1.3	
Fluor- Santamarine	0	1.57	0.35	0.67	1.37				
Alanto	0	0.03	0.01	0.03	1.33	1.33	1.33	1.33	
Alpha-Cyclo	0	0.15	0.15						
Beta-Cyclo	0	0.03	0.02	0.04	0.12				
3-DeBra	0	0.1	0.04	0.07	0.05	0.05	0.13		
APO	0	0.13	0.07	0.18	1.58				
DisOH	0	0.8	1.03	1.6	1.8				
DisNH2	0	1.31	0.95						
Fluor-APO	0	0.2	0.48	0.41	0.43				
Fluor-DisOH	0	1.16	1.29	1.65	1.71				
Fluor-DisNH	0	0.78	1.75						
DIBOAa	0	0.94	0.13	0.66	0.51	0.87			
DIBOAb	0	0.16	0.25	0.31	0.27	0.27			
DIMBOAa	0	1.45	0.71						
DIMBOAb	0	0.19	0.96	0.58	0.8	1.61	1.65		
DDIBOA	0	1.45	1.44	1.58	1.63	1.41			
6Cl-DDIBOA	0	0.11	0.09	0.06					
6F-DDIBOA	0	0.19	0.13	0.21					
8Cl-DDIBOA	0	0.18	0.72	0.68	0.57	0.7	0.38	0.52	

Table S3. RMSD values of tested compounds against Spike protein of SARS-CoV-2.

				**Spike SARS-CoV-2**						
**RMSD in Cluster**	**1**	**2**	**3**	**4**	**5**	**6**	**7**	**8**	**9**	
Azithromycin	0	1.52	0.91	0.81						
Hydroxychloroquine	0	1.88	0.67	0.73	0.41					
Favipiravir	0	0.36	0.49							
Artemisinin	0	0.07	0.11	0.18						
Met-4F-Benzo	0	0.87	1.07	0.45						
Cynaropicrin	0	1.84	1.95							
Fluor-Cynaro	0	0.99	0.43	0.09	0.09	0.08				
Costunolide	0	0.03	0.03	0.06						
DHC	0	0.52	0.1							
Reynosin	0	0.08	0.05	0.02	0.7	1.78				
Santamarine	0	0.15	1.01							
Fluor-Reynosin	0	0.76	1.56							
Fluor- Santamarine	0	0.83	0.54							
Alanto	0	0.01	0.02	0.08	0.08					
Alpha-Cyclo	0	0.01	0.01	0.08	0.06	1.91	1.35			
Beta-Cyclo	0	0.03	0.05	0.07	0.8					
3-DeBra	0	0.02	0.04	0.06	0.87	1.92				
APO	0	0.12	0.09							
DisOH	0	0.59	0.44							
DisNH2	0	1.71	1.78							
Fluor-APO	0	0.13	0.2	0.09	0.1					
Fluor-DisOH	0	1.96	0.69	0.72						
Fluor-DisNH	0	0.57	0.07							
DIBOAa	0	0.12	0.13	0.11	0.41					
DIBOAb	0	0.09	0.53	1.59						
DIMBOAa	0	0.28	1.99							
DIMBOAb	0	0.14	0.3	0.92	0.93	0.96	0.98	0.88	0.96	1.85
DDIBOA	0	0.08	0.19	1.25	1.6					
6Cl-DDIBOA	0	0.76	0.79							
6F-DDIBOA	0	1.49	1.86							
8Cl-DDIBOA	0	1.96	0.13							

Table S4. RMSD values of tested compounds against M^{pro} of SARS-CoV.

	M^{pro} SARS-CoV						
**RMSD in Cluster**	**1**	**2**	**3**	**4**	**5**	**6**	**7**
Azithromycin	0	1.19	0.99				
Hydroxychloroquine	0	1.11	0.13	1.01	1.99		
Favipiravir	0	0.19	0.45				
Artemisinin	0	0.02	0.07	0.04	0.05	0.12	1.11

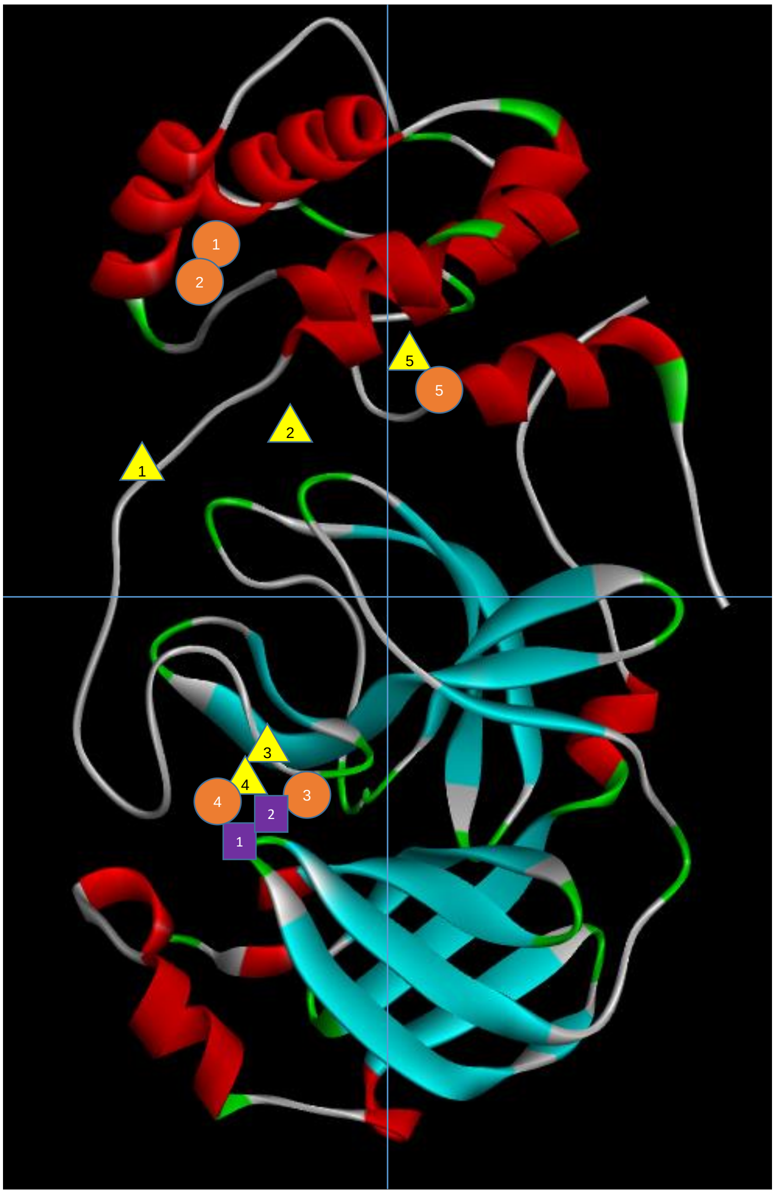

Figure S1. Bond mapping of M^{pro} with the most relevant ligand in the study. The image highlights that benzoxazinoids and sesquiterpenes explore a different binding site than the standards. The legend for the compounds is shown in the table below

Compounds	Nº	Symbol
Azythromycin	1	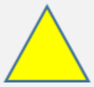
Hydroxychloroquine	2	
Favipiravir	3	
Artemisinin	4	
Met-4F-Benzo	5	
Costunolide	1	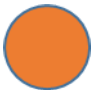
DHC	2	
Fluor-Reynosin	3	
Fluor-Santamarine	4	
3-DeBra	5	
Fluor-APO	1	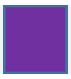
APO	2	

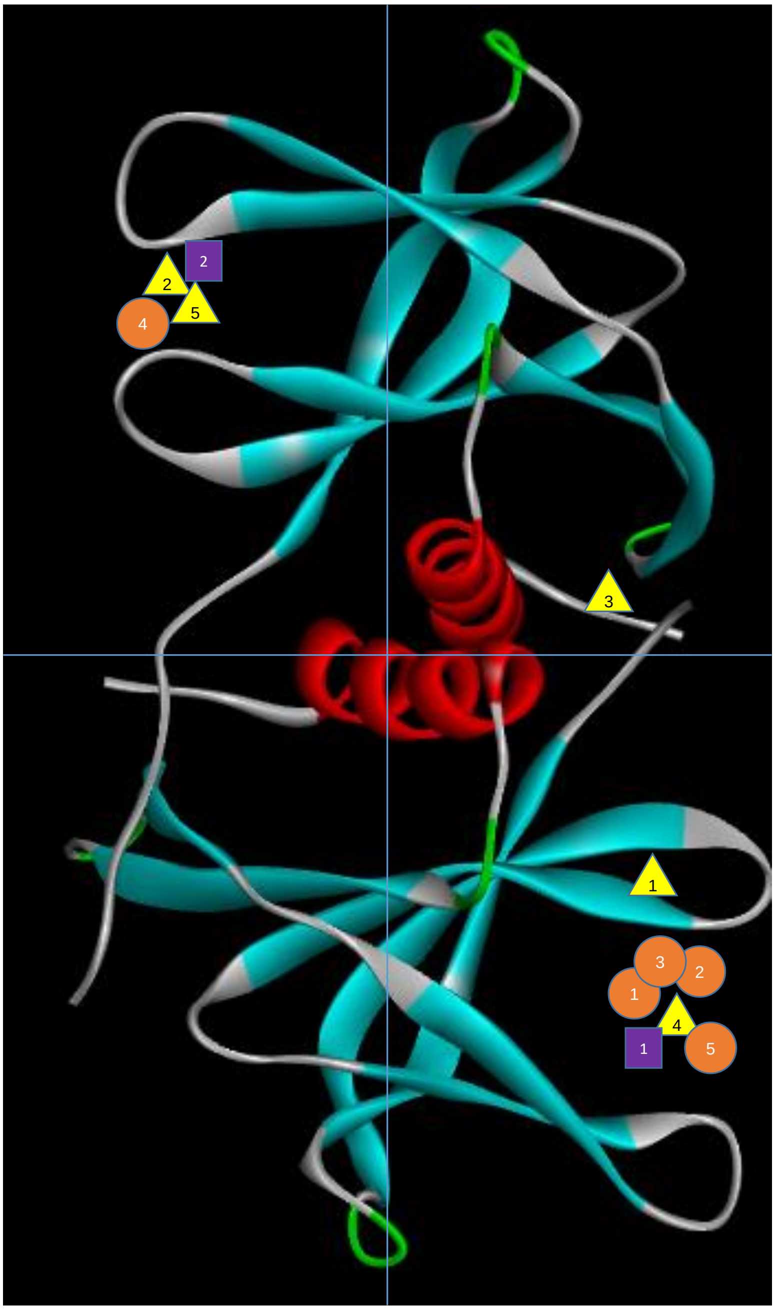

Figure S2. Bond mapping of the RNA replicase with the most relevant ligand in the study. In this case, the protein shows symmetry, so compounds in the first and fourth grid correspond to the same site.

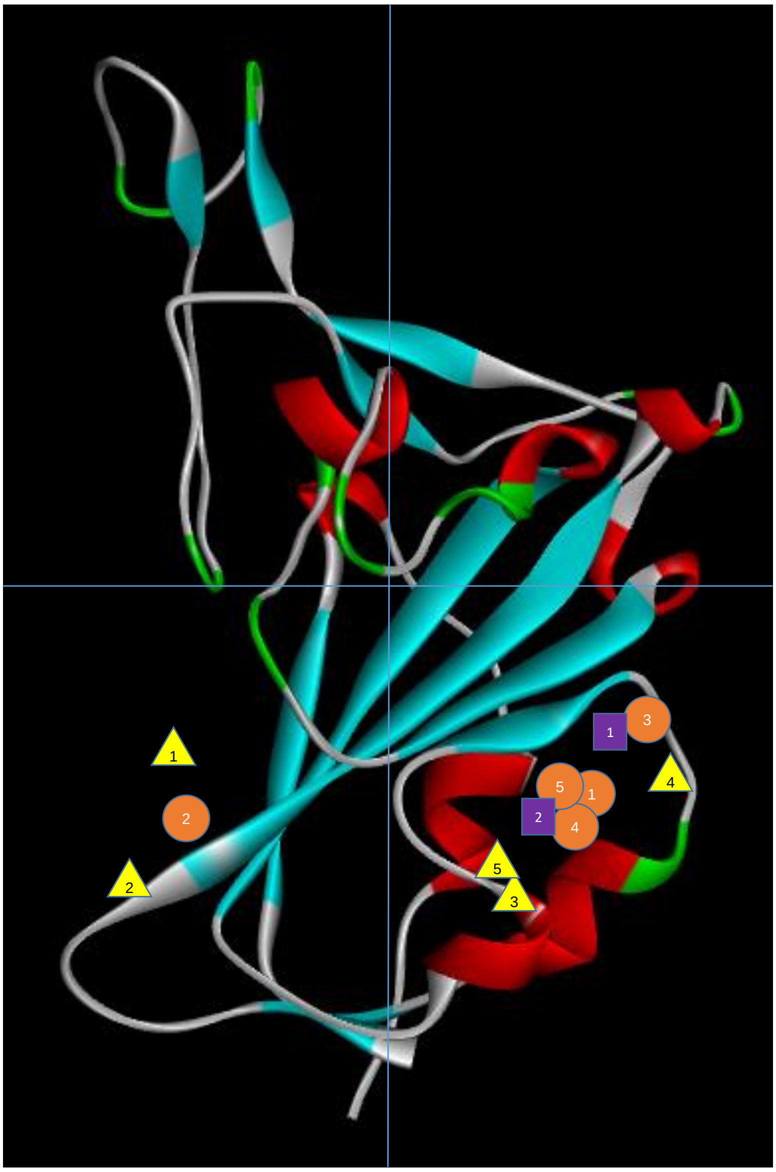

Figure S3. Bond mapping of the spike protein with the most relevant ligand in the study. The image shows that benzoxazinoids and sesquiterpenes explore different binding sites than the standards.

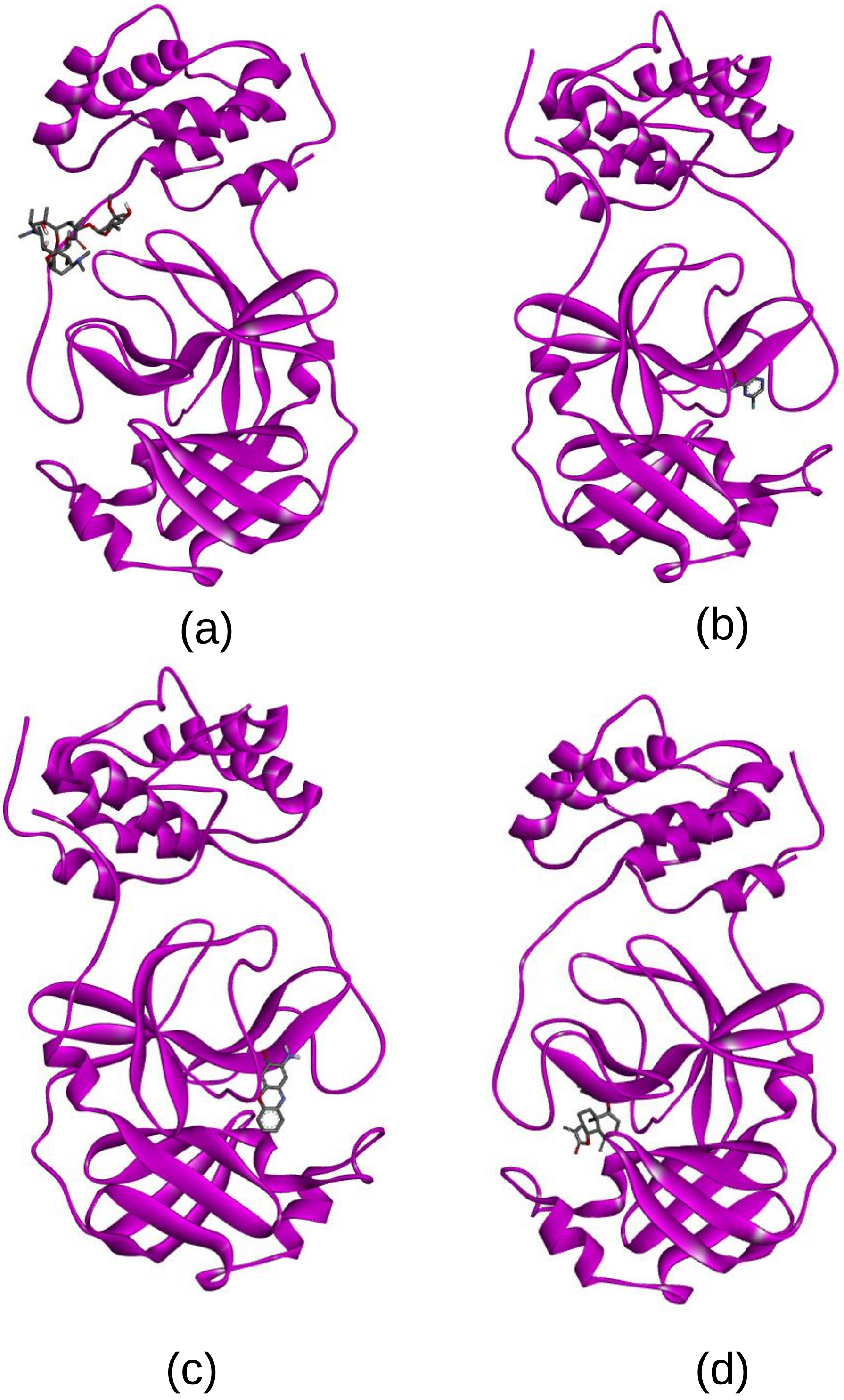

Figure S4. Comparative images of M^{pro} site of action of standard (**a**) azithromycin and (**b**) favipiravir, with (**c**) APO and (**d**) Fluor-Reynosin. (**a)** and (**b)** show different sites of action than (**c)** and (**d**). In the case of (**b**), the position is in front of the protein while (**c**) and (**d**) are behind. Sesquiterpenoids and benzoxazinoids explore a different kind of site.

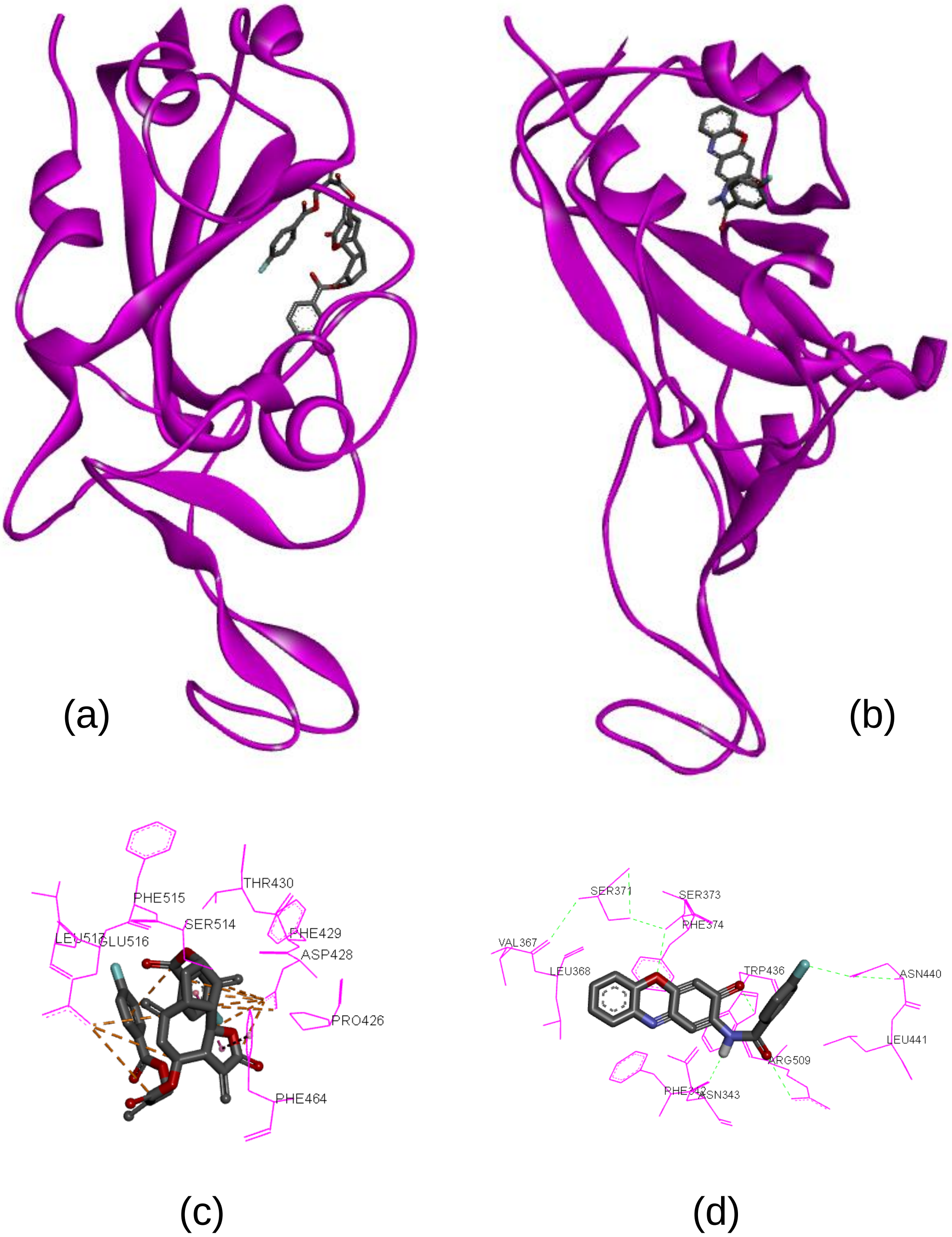

Figure S5. (**a**) Binding site of Fluor-Cynaro on the Spike protein of SARS-CoV-2. (**b**) Binding site of Fluor-APO on the Spike protein of SARS-CoV-2. (**c**) Amino acid residues that establish intermolecular forces with Fluor-Cynaro on the Spike protein of SARS-CoV-2. (**d**) Amino acid residues that establish intermolecular forces with Fluor-APO on the Spike protein of SARS-CoV-2.

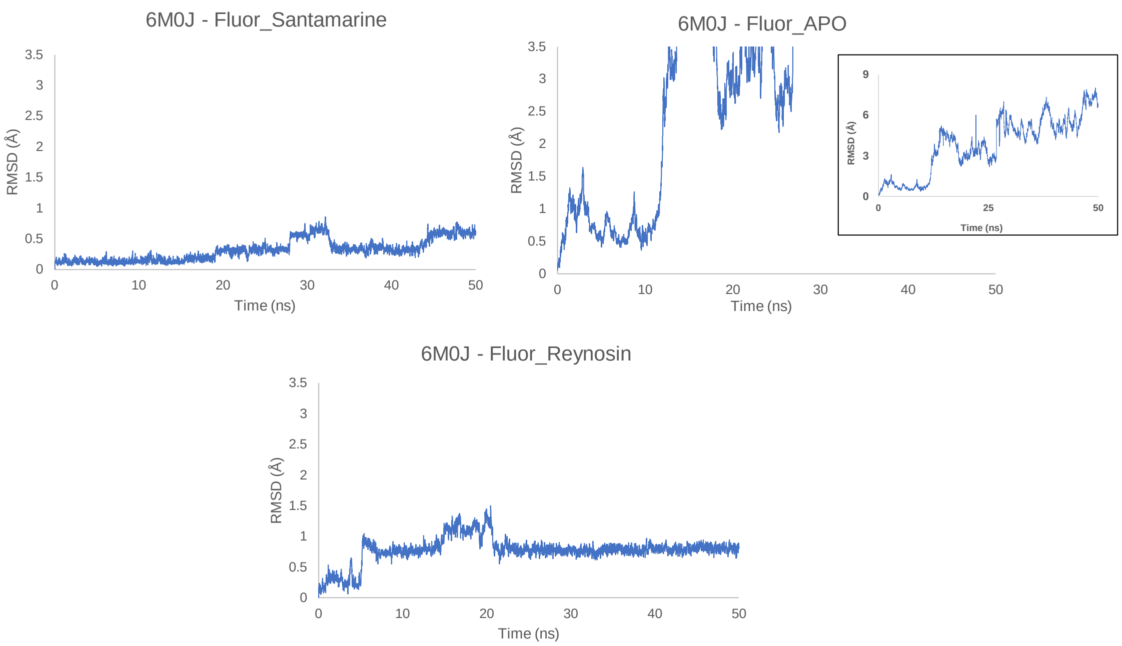

Figure S6. Root mean square deviation (RMSD) of the different ligands in protein-ligand complex with Spike protein of SARS-CoV-2.

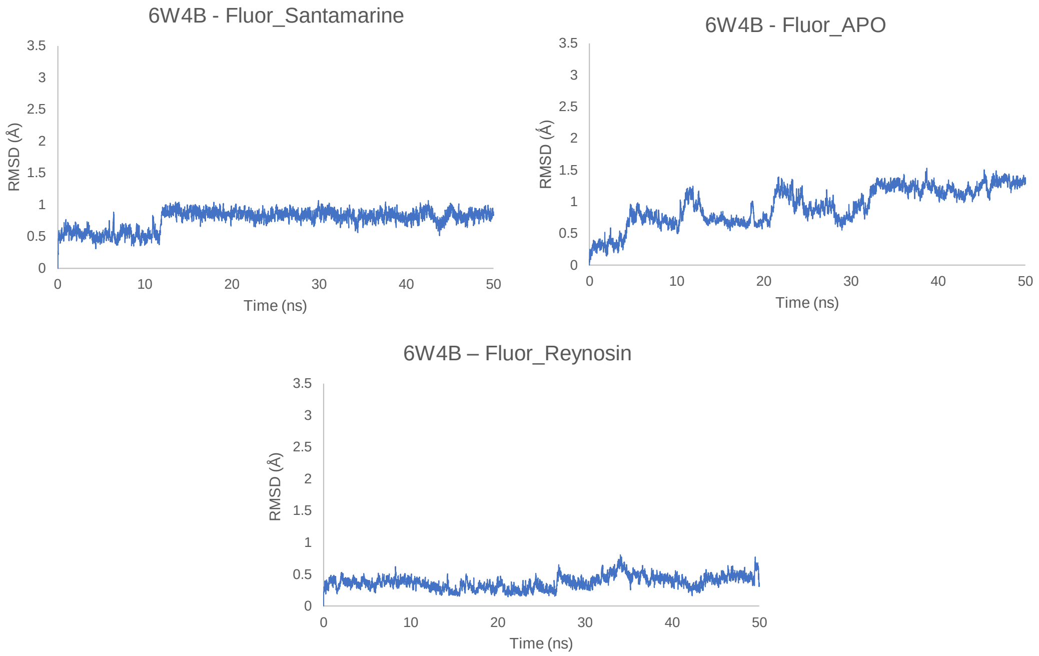

Figure S7. Root mean square deviation (RMSD) of the different ligands in protein-ligand complex with RNA replicase of SARS-CoV-2

Fluor-APO – 6LU7

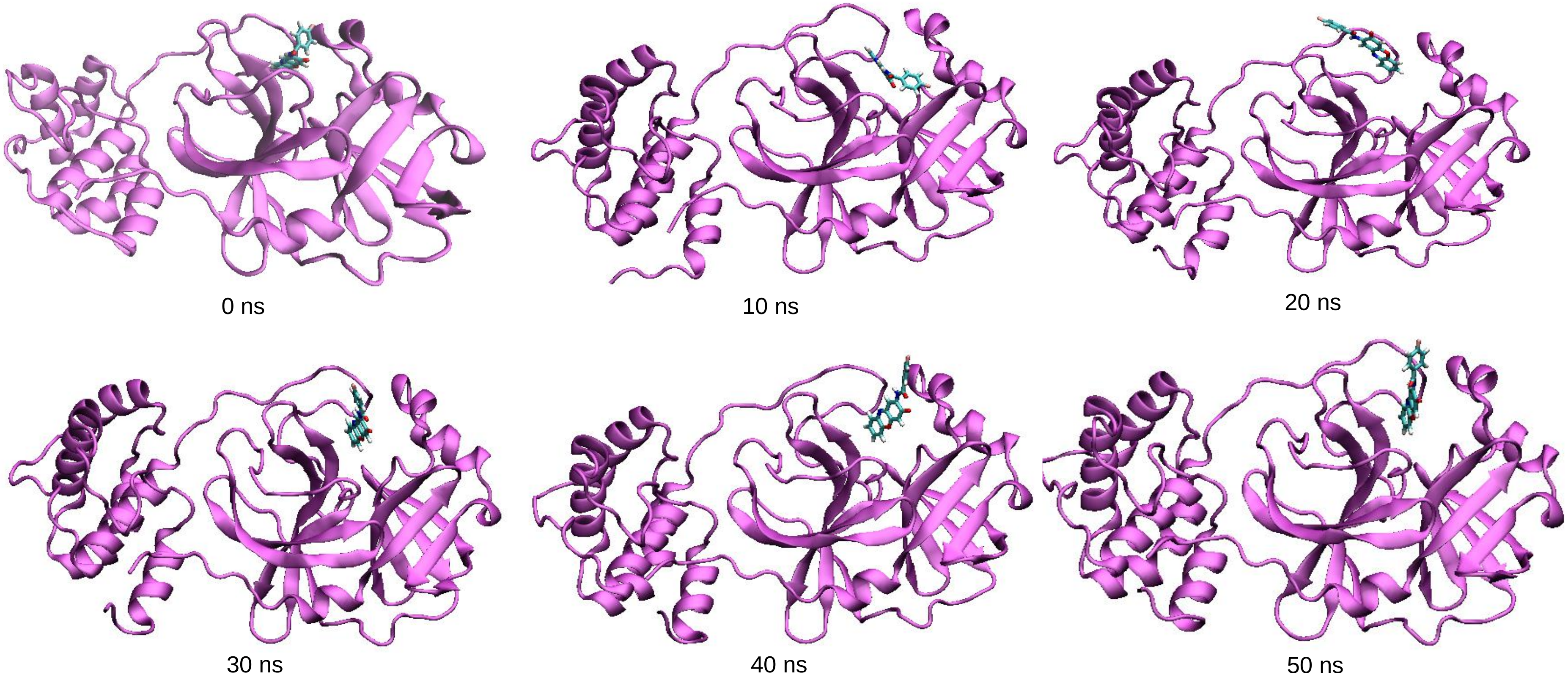

Figure S8. Snapshot of structural changes at different times of the molecular dynamics of Fluor-APO with main protease of SARS-CoV-2.

Fluor-Reynosin – 6LU7

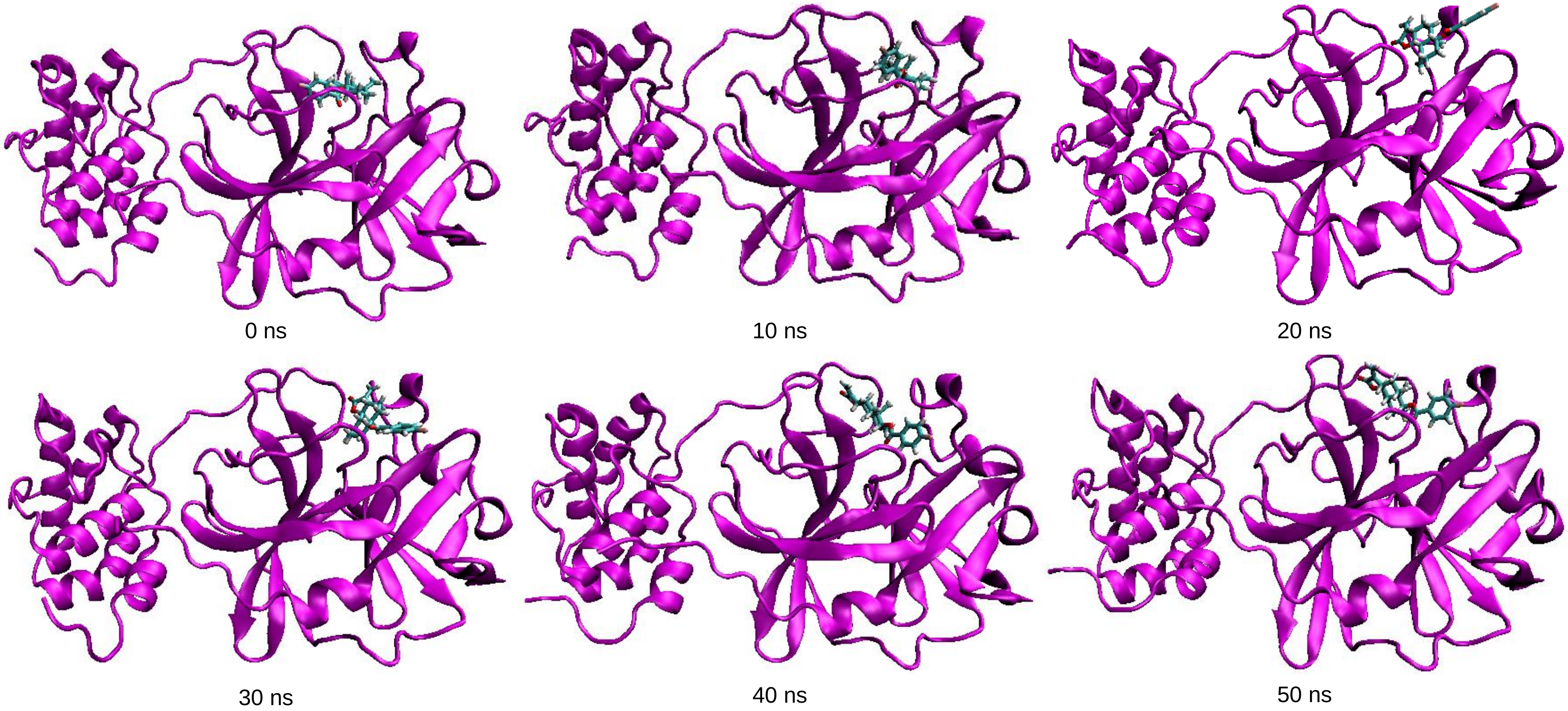

Figure S9. Snapshot of structural changes at different times of the molecular dynamics of Fluor-Reynosin with main protease of SARS-CoV-2.

Fluor-Santamarine – 6LU7

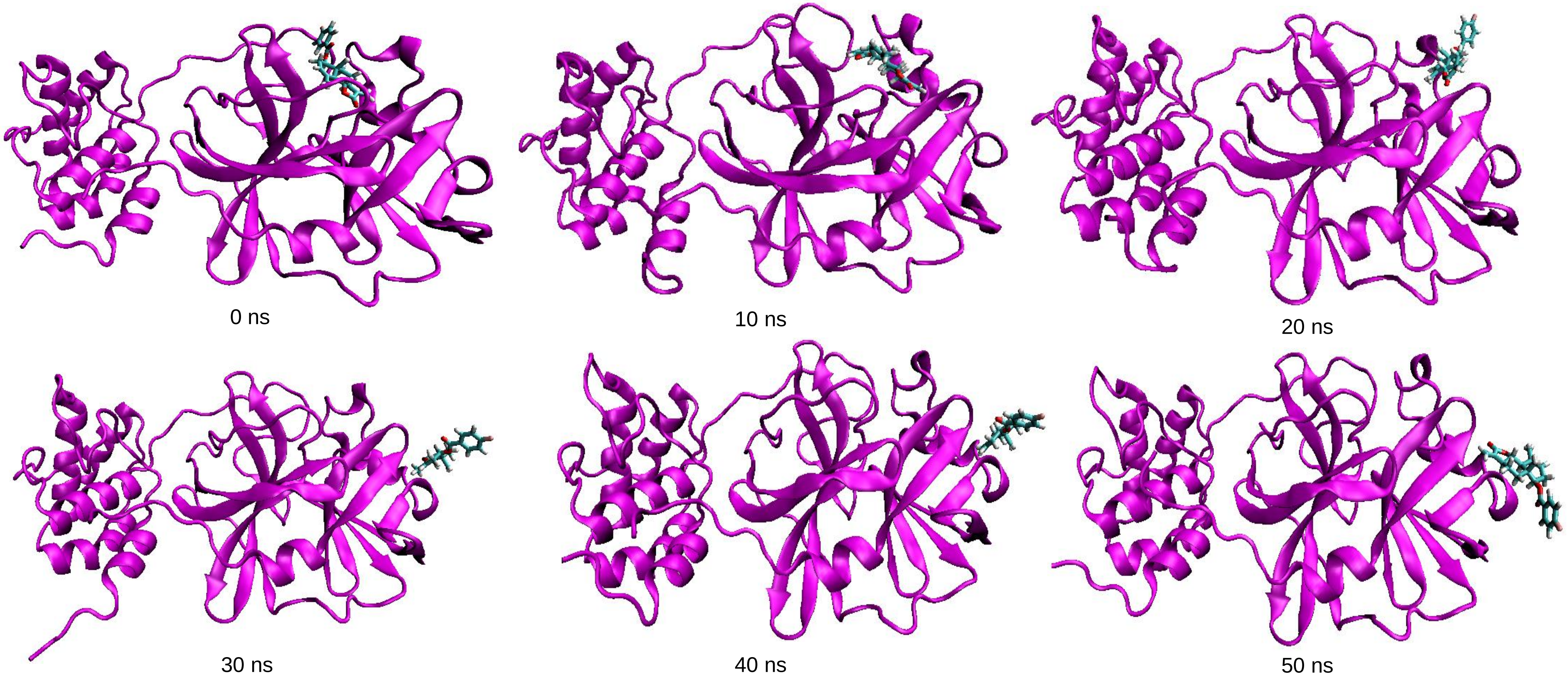

Figure S10. Snapshot of structural changes at different times of the molecular dynamics of Fluor-Santamarine with main protease of SARS-CoV-2.

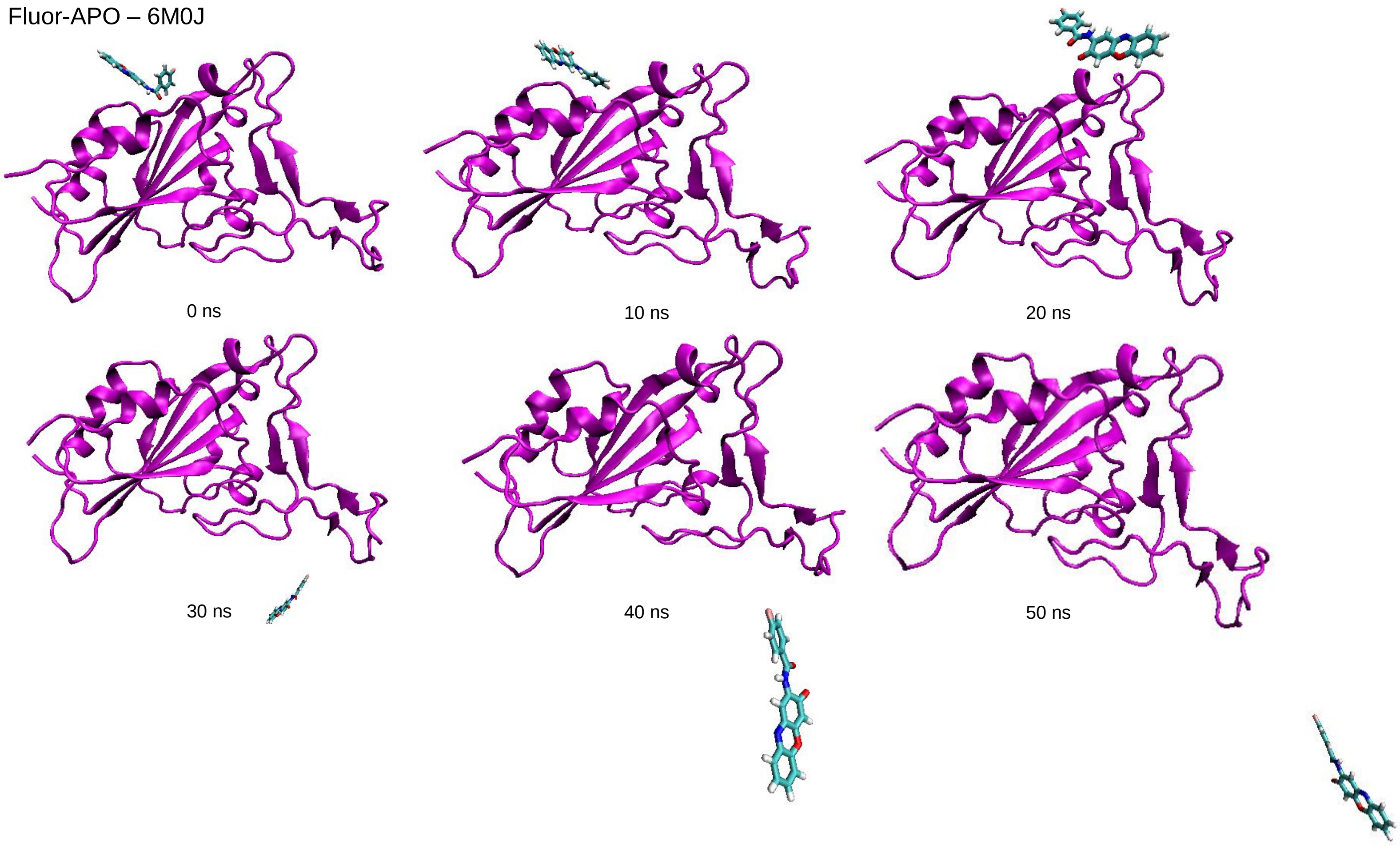

Figure S11. Snapshot of structural changes at different times of the molecular dynamics of Fluor-APO with spike protein of SARS-CoV-2.

Fluor-Reynosin – 6M0J

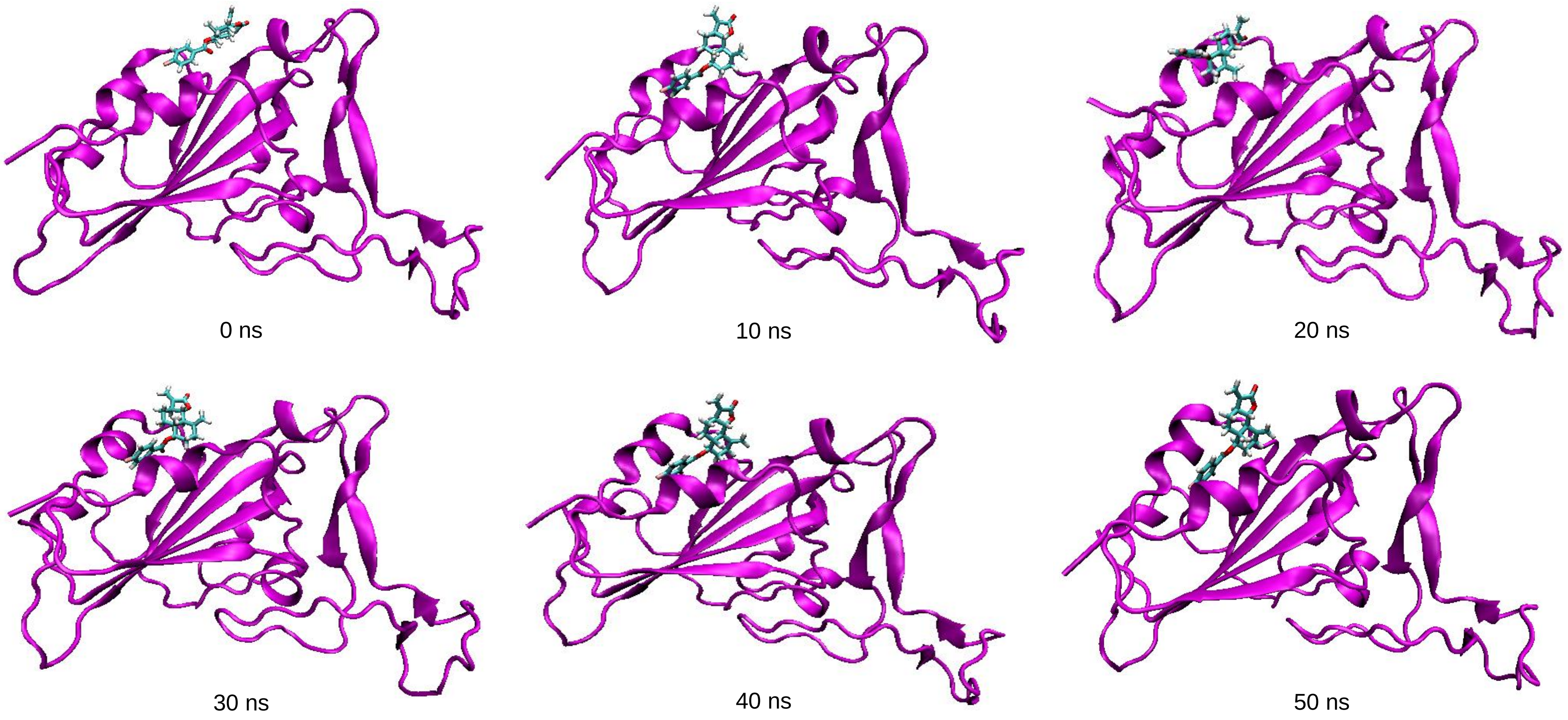

Figure S12. Snapshot of structural changes at different times of the molecular dynamics of Fluor-Reynosin with spike protein of SARS-CoV-2.

Fluor-Santamarine – 6M0J

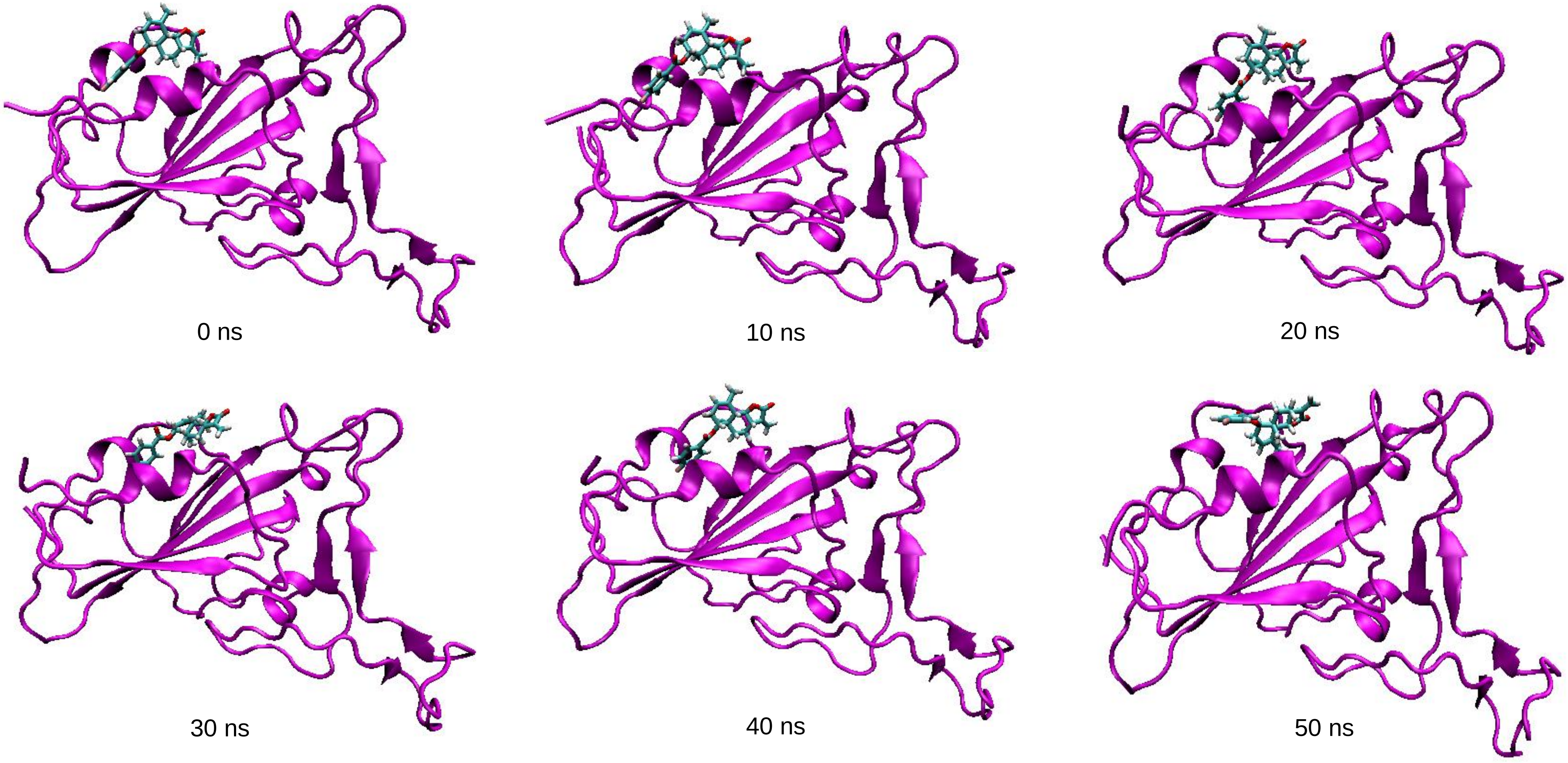

Figure S13. Snapshot of structural changes at different times of the molecular dynamics of Fluor-Santamarine with spike protein of SARS-CoV-2.

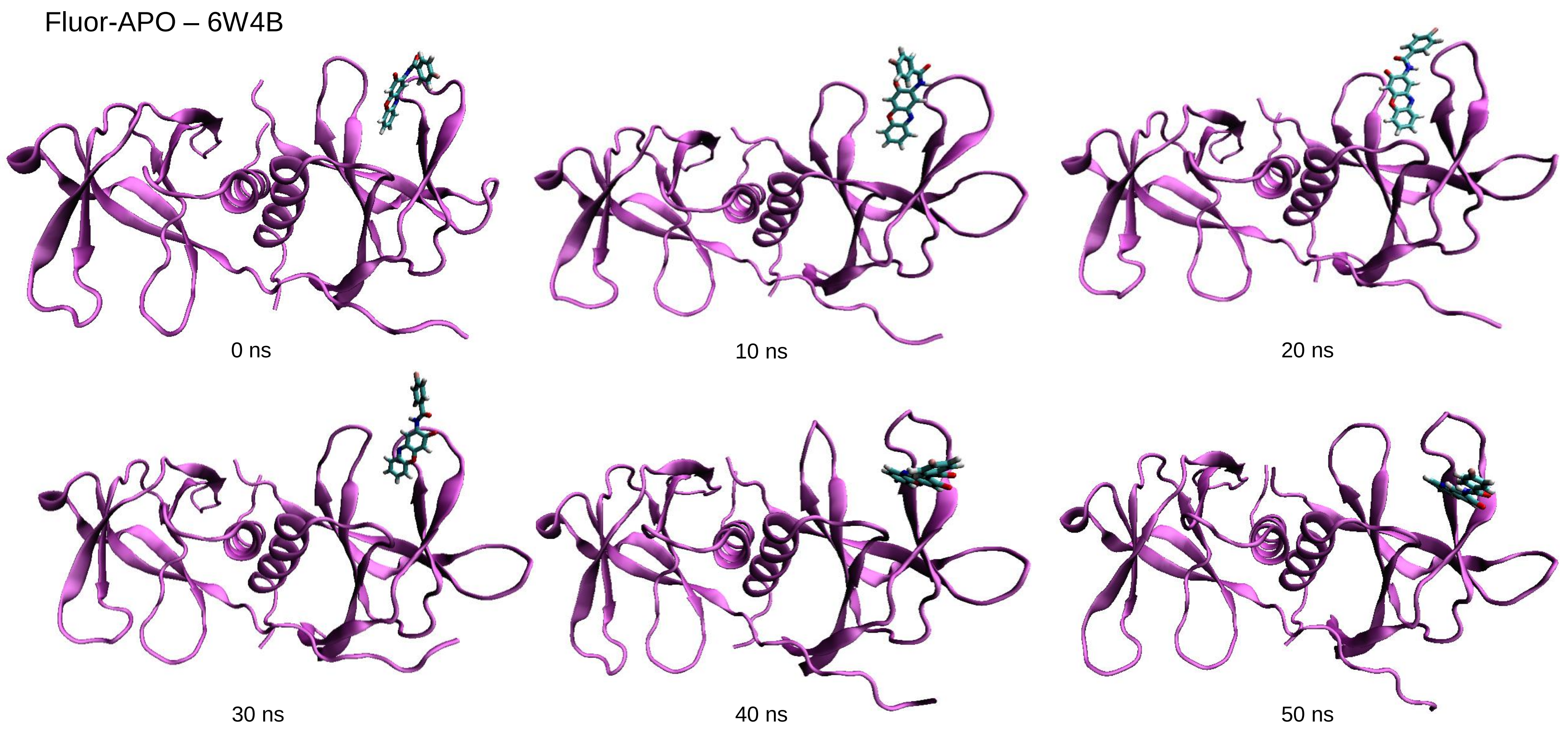

Figure S14. Snapshot of structural changes at different times of the molecular dynamics of Fluor-APO with RNA replicase of SARS-CoV-2.

Fluor-Reynosin – 6W4B

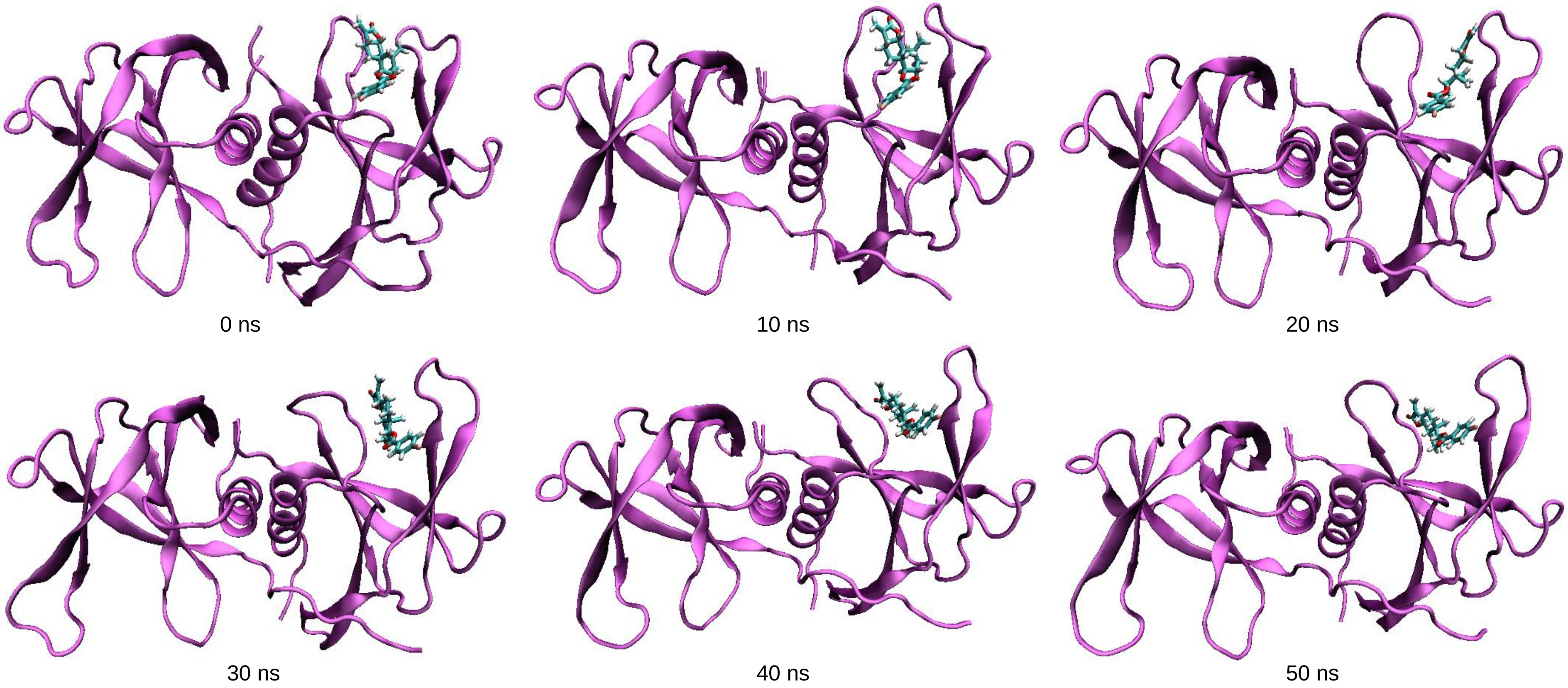

Figure S15. Snapshot of structural changes at different times of the molecular dynamics of Fluor-Reynosin with RNA replicase of SARS-CoV-2.
